# Exploration of chlorophyll fluorescence characteristics gene regulatory in rice (*Oryza sativa* L.): a genome-wide association study

**DOI:** 10.3389/fpls.2023.1234866

**Published:** 2023-09-07

**Authors:** Sicheng Liu, Zhuang Xiong, Zuolin Zhang, Youbo Wei, Dongliang Xiong, Fei Wang, Jianliang Huang

**Affiliations:** ^1^ Ministry of Agriculture Key Laboratory of Crop Ecophysiology and Farming System in the Middle Reaches of the Yangtze River, College of Plant Science and Technology, Huazhong Agricultural University, Wuhan, China; ^2^ Institute of Food Crops, Hubei Academy of Agricultural Sciences, Wuhan, China

**Keywords:** rice, chlorophyll content, fluorescence parameters, GWAS, candidate gene, transcriptome

## Abstract

Chlorophyll content and fluorescence parameters are crucial indicators to evaluate the light use efficiency in rice; however, the correlations among these parameters and the underlying genetic mechanisms remain poorly understood. Here, to clarify these issues, we conducted a genome-wide association study (GWAS) on 225 rice accessions. In the phenotypic and Mendelian randomization (MR) analysis, a weak negative correlation was observed between the chlorophyll content and actual quantum yield of photosystem II ( ${\text{Φ}}_{II}$ ). The phenotypic diversity observed in *SPAD*, $NPQ_{t}$ , ${\text{Φ}}_{NPQ}$ , and $F_{v}/F_{m}$ among accessions was affected by genetic background. Furthermore, the GWAS identified 78 SNPs and 17 candidate genes significantly associated with *SPAD*, $NPQ_{t}$ , ${\text{Φ}}_{II}$ , ${\text{Φ}}_{NPQ}$ , $q_{L}$ and $q_{P}$ . Combining GWAS on 225 rice accessions with transcriptome analysis of two varieties exhibiting distinct fluorescence characteristics revealed two potential candidate genes (*Os03g0583000* from ${\text{Φ}}_{II}$ & $q_{P}$ traits and *Os06g0587200* from $NPQ_{t}$ trait), which are respectively associated with peroxisomes, and protein kinase catalytic domains might involve in regulating the chlorophyll content and chlorophyll fluorescence. This study provides novel insights into the correlation among chlorophyll content and fluorescence parameters and the genetic mechanisms in rice, and offers valuable information for the breeding of rice with enhanced photosynthetic efficiency.

## 1 Introduction

Promotion of food security is crucial with the increase in global population and decrease in arable land (West et al., 2014). Rice (*Oryza sativa* L.) is the staple food for over half of the world’s population (Nguyen, 2002; Bandumula et al., 2018). Photosynthesis is the key determinant of rice yield, as it is the most crucial process that influences the biomass accumulation and harvest index (Makino, 2011; Ambavaram et al., 2014). In the past decades, the Green Revolution has effectively increased rice yield by enhancing the lodging resistance and harvest index (Khush, 2001; Liu et al., 2021). However, these improvements have reached their limits. Recent studies have indicated that the current light use efficiency of rice is significantly lower than its biological potential, suggesting that mediation of light use efficiency is a promising way to further enhance rice productivity (Zhu et al., 2008; Long et al., 2015). In recent years, phenotype selection and hybrid breeding aimed at improving the light use efficiency have greatly enhanced rice yield, contributing to significant improvement of rice production efficiency and food security (Song et al., 2010; Qu et al., 2017).

Leaves are primary organs of photosynthesis, and the photosynthetic capacity can be assessed using indicators such as chlorophyll content, chlorophyll fluorescence, and gas exchange parameters. Compared with gas exchange parameters, chlorophyll content and fluorescence parameters have the advantages of simplicity, speed, and high throughput (Bolharnordenkampe et al., 1989). Among them, chlorophyll content in leaves can indicate leaf photosynthetic capacity, and is positively correlated with the photosynthetic rate (Fleischer, 1935; Kurahotta et al., 1987; Croft et al., 2017). Chlorophyll fluorescence parameters are also closely associated with plant photosynthesis and are widely used in both *in vivo* and *in vitro* studies of plant photosynthesis (Baker, 2008). Non-photochemical quenching (*NPQ*) (Genty et al., 1989), ${\text{Φ}}_{NPQ}$ (Kuhlgert et al., 2016), and $NPQ_{t}$ (Tietz et al., 2017) can describe the excited state of chlorophyll a, which is a major and extensively studied photoprotective mechanism for plants to survive under high light conditions (Demmig-Adams et al., 2014). Kromdijk et al. (2016) found that acceleration of the xanthophyll cycle leads to rapid *NPQ* recovery, thereby increasing plant carbon assimilation efficiency. Kohzuma (2019) revealed significant differences in the light-dependent changes in *NPQ* and the photochemical reflectance index between the wild type and *npq1* mutant. Actual quantum yield of photosystem II ( ${\text{Φ}}_{II}$ ) (Genty et al., 1989) is a crucial indicator of photosynthetic efficiency, and increasing the thylakoid density with nitrogen can improve the quantum yield by enhancing the overall light absorption. Hogewoning et al. (2012) demonstrated that the quantum yield of plants can be affected by the light with different wavelengths. Furthermore, chlorophyll content and fluorescence parameters can reflect the response of total photosynthetic productivity to environmental factors, such as temperature (Mishra et al., 2014), dehydration (Banks, 2018), and nutrient deficiency (Ciompi et al., 1996; Feng et al., 2015). Flag leaves serves as the most significant source of organ and plays a dominant role in providing assimilates for grain development (Li et al., 1998). And flag leaves shares the same genetic system with other leaves, making it a representative of leaf characteristics to a certain extent (Yin et al., 2017). Therefore, exploring the genetic factors controlling the chlorophyll content and fluorescence parameters in the flag leaves of rice is crucial for enhancing the photosynthetic productivity and yield of rice.

Genome-wide association study (GWAS) is a powerful tool for identifying genetic variations. When combined with other methods such as transcriptomics and Mendelian randomization (MR) (Sanderson et al., 2022), GWAS can be used to identify critical genes to develop crops with higher photosynthetic capacity and grain yield. Rice is one of the most extensively studied crops, and many projects such as the 3K Rice Genomes Project have provided extensive genetic data for the research (Wang et al., 2018). Proper distribution of photosynthetic energy can improve the efficiency of crop light use efficiency, and some studies have identified the functional genes related to chlorophyll content (Wang et al., 2015), fluorescence parameters (Hao et al., 2012; Wang et al., 2017), and photosynthesis (Wang et al., 2020; Miao et al., 2023) through genetic mapping and natural population identification, which can greatly facilitate the research on plant productivity and food security. However, few loci or genes in these natural variations have been reported to be involved in the genetic basis of rice chlorophyll content, fluorescence parameters, and their interrelationships, and there has been a lack of comprehensive and effective analysis of the genetic basis and relationship of these traits.

In this study, we identified 78 SNPs related to chlorophyll fluorescence characteristics through genotype screening and GWAS based on the phenotypes of 225 rice accessions, including chlorophyll content (*SPAD*) and eight fluorescence parameters ( $NPQ_{t}$ , ${\text{Φ}}_{II}$ , ${\text{Φ}}_{NO}$ , ${\text{Φ}}_{NPQ}$ , LEF, $F_{v}/F_{m}$ , $q_{L}$ and $q_{P}$ ). A negative phenotypic correlation was observed between ${\text{Φ}}_{II}$ and *SPAD*. Mendelian randomization (MR) analysis was employed to further estimate the genetic relationship between ${\text{Φ}}_{II}$ and *SPAD*. In addition, we conducted a transcriptome analysis on two varieties with significant differences in phenotype, and identified 2,366 differentially expressed genes (DEGs), as well as the key regulatory genes and pathways. Finally, by combining GWAS, transcriptome analysis, gene annotation, and GO analysis, we identified two candidate genes (*Os03g0583000* and *Os06g0587200*) related to rice chlorophyll fluorescence characteristics. This study lays a foundation for future research on phenotypic screening, gene function verification, genetic mechanism dissection, and genetic enhancement of rice chlorophyll fluorescence characteristics and photosynthesis.

## 2 Materials and methods

### 2.1 Plant materials and field experiment

The study was conducted using a diverse collection of 225 *Oryza sativa* accessions, consisting of 83 accessions from the Mini Core Collection of Huazhong Agricultural University and 142 accessions from the 3K Rice Genomes Project. These accessions originated from various parts of the world and encompassed different subpopulations, which can complete their reproductive cycle in Wuhan. **Table S1** provides the details of the accessions, including their names and countries of origin.

The experiment was conducted in the field of Huazhong Agricultural University, Wuhan, China. About 200 g of seeds were sown on May 15^th^ of 2018 and May 18^th^ of 2019. 30-day-old seedlings were transplanted into 1 m × 2 m plots, with one plant per hill at a spacing of 0.20 × 0.25 m. Fertilizers applied to all plots were 180 kg N ha^-1^, 60 kg P_2_O_5_ ha^-1^, and 120 kg K_2_O ha^-1^. The plots received standard management practices, including irrigation, fertilization, and disease and pest control. **Figure S1** shows the weather data for the whole growing season.

To unify the data from two years, we used the lmer function within the lme4 package. The phenotype data were modeled with a linear mixed model, where accession was the fixed effect and year and replication were the random effects, to calculate the BLUE (best linear unbiased estimator, fixed factor) values to be used in the GWAS analysis. The following formula was used to calculate the heritability:


(1) $$\text{Heritability}=\;\frac{V_{G}}{V_{G}+\;\frac{1}{e}\;V_{GE}+\;\frac{1}{re}V_{\text{ϵ}}}$$

, where, $V_{G}$ , $V_{GE}$ , $V_{\text{ϵ}}$ , r , and e represent the genetic variance, interaction variance between genotypes and environments, error variance, number of replicates within each environment, and number of environments, respectively. Data entry was done using MS Office, while analysis and processing were carried out using the R software (https://cran.r-project.org/).

### 2.2 *SPAD* and chlorophyll fluorescence measurements

Five plants of each accession in the middle of the plot were selected to investigate the chlorophyll content and fluorescence characteristics at the heading stage. Chlorophyll content and fluorescence parameters were measured in the middle part (1/3~2/3) of the flag leaves between 8:30 and 11:30 a.m. on a sunny day, using a portable chlorophyll fluorometer (MultispeQ v1.0) to obtain more reliable data in the field setting. The instrument was used with the protocol “Leaf Photosynthesis MultispeQ V1.0 no open/close” provided at https://www.photosynq.org/protocols/leaf-photosynthesis-multispeq-v1-0-no-open-close, which is a classic and by far the most utilized PhotosynQ Protocol for measuring many photosynthesis-related parameters in a short period of time. Due to insufficient dark adaptation during the measurement, our $F_{v}/F_{m}$ parameter is not rigorous and can only reflect the plant’s state at the time of measurement. The *SPAD* of the leaf was calculated by measuring the absorbance at 650 nm and 940 nm, and k is the calibration coefficient obtained using MultispeQ calibration cards.


(2) $$SPAD\;=\;k\times \;log_{10}\left(\frac{Abs940nm/ref.Abs940nm}{Abs650nm/ref.Abs650nm}\right)$$

The eight fluorescence parameters were calculated based on the minimum fluorescence ( $F_{o}$ ), maximum fluorescence ( $F_{m}$ ), steady-state fluorescence ( $F_{s}$ ), $F_{o}^{'}$ and $F_{m}^{'}$ (the same as above but measured under light conditions), and photosynthetically active radiation (PAR). The calculation of ${\text{Φ}}_{II}$ , ${\text{Φ}}_{NO}$ , ${\text{Φ}}_{NPQ}$ , and $F_{v}/F_{m}$ parameters related to photosynthetic efficiency was carried out as follows:


(3) $${\text{Φ}}_{II}\;=\;\frac{F_{m}^{'}-F_{s}}{F_{m}^{'}}\;$$

(4) $${\text{Φ}}_{NO}\;=\;\frac{F_{s}}{F_{m}}$$

(5) $${\text{Φ}}_{NPQ}=1-{\text{Φ}}_{II}-{\text{Φ}}_{NO}$$

(6) $$F_{V}/F_{m}\;=\;\frac{F_{m}-F_{o}}{F_{m}}$$

The linear electron flow (LEF) was calculated as follows:


(7) $$LEF=\;{\text{Φ}}_{II}\;\times PAR\times 0.4$$

The $q_{L}$ and $q_{P}$ , which reflect the “Lake” model and “Puddle” model in Photosystem II Redox State, was calculated as follows:


(8) $$q_{P}\;=\;\frac{F_{m}^{'}\;-\;F_{s}}{F_{m}^{'}\;-\;F_{o}^{'}}$$

(9) $$q_{L}\;=\;q_{P}\;\times \;\frac{F_{o}^{'}}{F_{s}}$$

$NPQ_{t}$ , an efficient parameter that reflects NPQ, was calculated without the need for complete relaxation of the quenching process. The calculation for $NPQ_{t}$ was as follows:


(10) $$NPQ_{t}=\left(\frac{4.88}{\frac{F_{m}^{'}}{F_{o}^{'}}-1}\right)-1$$

### 2.3 DNA isolation, sequencing, and data processing

DNA was extracted from fresh leaves of field-grown plants using a modified CTAB method (Yan et al., 2008). Whole-genome DNA sequencing was performed on the Illumina HiSeq-2000 platform by Personalbio (Shanghai, China). (Andrews, 2010) (V0.11.9) was used for quality control of sequencing data, and paired-end 150 bp reads were mapped to the Nipponbare reference genome (https://www.ebi.ac.uk/ena/data/view/GCA_001433935.1) using BWA (V0.7.17) with the default parameters. After alignment, the genomic data were sorted using SAMtools (V1.9) and the sequencing reads were de-duplicated using SAMBAMBA (V0.8.2). Genomic variants (in GVCF format for each accession) were identified using the Genome Analysis Toolkit (GATK V4.3.0) software, with the HaplotypeCaller module and GVCF model. The raw variant sites were further filtered by Plink (V1.9), with genotype quality for each individual ≥ 10%. After genotype imputation using Beagle (V4.1), the minor allele frequency (MAF) was controlled to be ≥ 5%. The identified SNPs were further annotated using the ANNOVAR software (version 16-Jul-2017).

### 2.4 GWAS analysis

All 442,634 identified SNPs were used to build a phylogenetic tree and perform principal component analysis (PCA). The individual-based neighbor-joining (NJ) tree was constructed using the phylip (V3.697) and EvolView (http://www.evolgenius.info/), based on the p-distance and with 1,000 bootstrap replicates. PCA was conducted using the Plink (V1.9) with the command “–pca 10” to output the top 10 PCA results. Since the first three principal components are more representative, we utilized the top three PCA results in the subsequent GWAS analysis. To estimate the LD in our rice population, the squared correlation coefficient (*r^2^*) between pairwise SNPs was computed using PopLDdecay (Zhang et al., 2019). The *r^2^* value was calculated for pairwise markers in a 1000-kb window and averaged across the whole genome. The “–cv” command of Admixture (V1.3.0) was used to calculate the cross-validation error for K = 2, 3, 4, and 5.

GWAS was performed using a mixed linear model (MLM) in the GEMMA (V0.98.1) package (Zoubarev et al., 2012). The matrix of pairwise genetic distances calculated by GEMMA was used as the variance-covariance matrix of random effects. The kinship matrix kin.sXX.txt was calculated using the command “-gk 2 -p Phenotype” and GWAS analysis was conducted using the command “-k kin.sXX.txt -lmm 1 -p Phenotype -c PCA”. Significant p-value thresholds P< 1.13 × 10^-7^ (0.05/442,634) were set to control the genome-wide type 1 error rate, which was calculated by 0.05/n (total SNPs). PVE of 100 kb was filtered out before and after the peak signal. The Manhattan and quantile-quantile (QQ) plots of GWAS results were generated in R software (https://cran.r-project.org/).

### 2.5 MR analysis

To consistently estimate the genetic effect of ${\text{Φ}}_{II}$ and *SPAD*, the genetic variants were selected according to the three assumptions in MR analysis, (i) the genetic variants were obtained from the results of GWAS associated with the single component trait at a genome-wide significant level (P < 1.13 × 10^-7^); (ii) the genetic variants were not associated with any confounders; (iii) the genetic variants only affected *SPAD* through the ${\text{Φ}}_{II}$ trait, not through other component traits (P > 0.05).

The MR Egger, Weighted Median, Inverse Variance Weighted, Simple Mode and Weighted Mode methods were used for MR analysis to assess the effect of ${\text{Φ}}_{II}$ on *SPAD*, by summarizing the effects of multiple independent SNPs. In sensitivity analysis, the MR Egger method and Inverse Variance Weighted method were used for MR analysis. According to the results, leave-one-out analysis was supplemented. MR analysis was performed in R package TwoSampleMR (https://mrcieu.github.io/TwoSampleMR/).

### 2.6 RNA isolation and candidate gene expression analysis

Total RNA was separately extracted from each sample using an RN38 EASYspin plus Plant RNA kit (Aidlab Biotech, Beijing, China). RNA integrity was determined through the RNA Nano 6000 Assay Kit of the Bioanalyzer 2100 system (Agilent Technologies, CA, United States). The libraries were sequenced by Personalbio (Shanghai, China) with an Illumina HiSeq (Illumina, CA, United States) system. To ensure the accuracy, reads with more than 10% N bases and low-quality reads with Q ≤ 20 and over 50% bases were excluded (Chen et al., 2018). The resulting clean reads were mapped to the Nipponbare reference genome using Tophat2 (Kim et al., 2013). Gene expression was then calculated by counting the number of mapped clean reads for each gene normalized into Fragments Per Kilobase of transcript sequence per Millions (FPKM).

### 2.7 Differential gene expression and functional enrichment analysis

DESeq2 R package (Love et al., 2014) was used for multiple testing correction of DEGs, and the false discovery rate (FDR) was calculated through the Benjamini and Hochberg’s method. DEGs were defined as genes exhibiting at least a 2-fold difference in expression, with |log2FoldChange(L2FC)| > 1, and P < 0.05. The Pheatmap R package (https://www.rdocumentation.org/packages/pheatmap/) performs bidirectional clustering analysis on the union of all DEGs and samples in all comparison groups. The clusterProfiler R package (Yu et al., 2012) was used to perform GO enrichment analysis for DEGs, with the p-value adjusted through the Benjamini and Hochberg’s method and a P < 0.05 selected as the threshold for determining significant GO terms. For all samples, PCA was carried out to explain their interrelationship. Blast2GO (Conesa et al., 2005) was used for DEG annotation and functional prediction.

## 3 Results

### 3.1 Genomic variation and population structure

The filtering generated a total of 632.17 GB of high-quality reads, which were mapped to the Nipponbare reference genome, with an average success rate of 94.4% (**Table S2**) and an average sequencing depth of 16.5-fold (**Table S2**). A total of 9,989,556 SNPs were identified on 12 chromosomes from the mapping, with the highest and lowest density of SNPs being detected on chromosome 11 and chromosome 03, respectively, and the average marker density was 27.10 SNPs/kb (**Table S3**). After filtering out SNPs with a low genotyping rate using PLINK, gene imputation was performed using Beagle. A final set of 442,634 SNP markers with a MAF greater than 0.05 was retained for GWAS analysis (**Table S4**).

**Figure 1:**
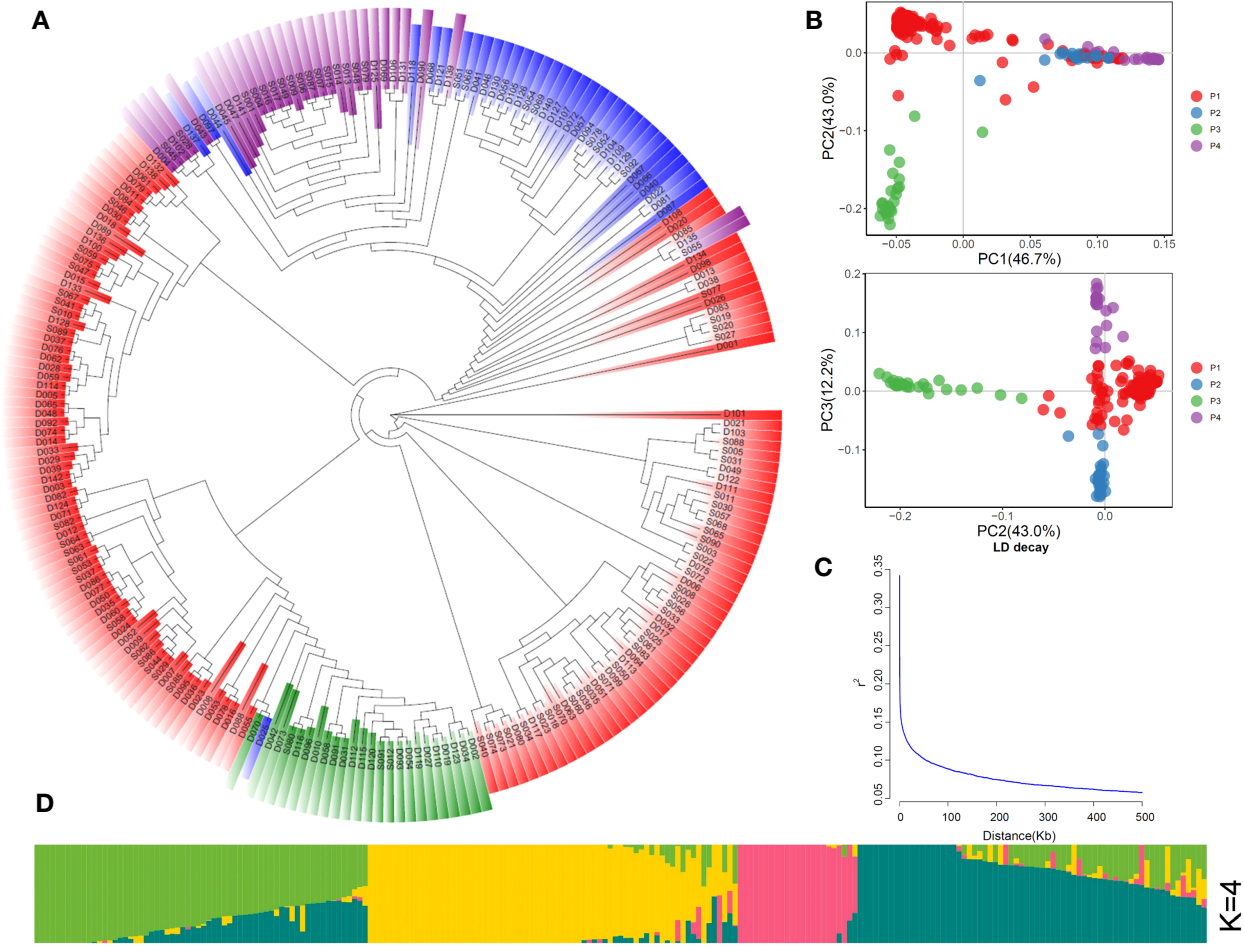
Population structure of 225 rice accessions. **(A)** Neighbor-joining phylogenetic tree. **(B)** PCA plots of the first three components. **(C)** Genome-wide average linkage disequilibrium (LD) decay. **(D)** Inferred membership fractions of the genotypes in sub-populations (K = 4).

PCA (**Figure 1B**) divided the population into four groups, which is consistent with the results of phylogenetic tree (**Figure 1A**), and the K value was considered as the number of subgroups with the lowest cross-validation (CV) error (**Figures 1D**, **S2**). **Figure 1C** shows the average linkage disequilibrium (LD) decay in the whole genome. **Figure 1** indicated that these materials could be divided into four groups with some genetic differences from each other.

### 3.2 Phenotypic analysis of chlorophyll fluorescence characteristics

**Figure 2:**
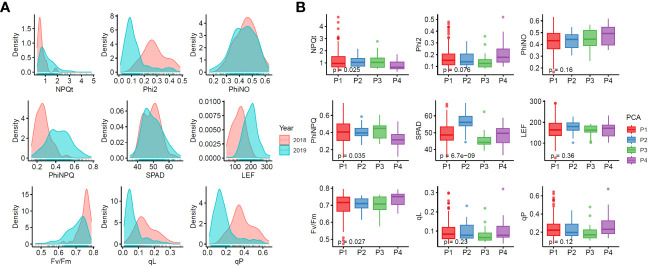
Phenotypic variations of chlorophyll fluorescence characteristics in 225 rice accessions. **(A)** Density distribution graphs for each trait. **(B)** Boxplots of chlorophyll fluorescence characteristics in four PCA groups. Phi2, PhiNO, and PhiNPQ respectively represent ${\text{Φ}}_{II}$ , ${\text{Φ}}_{NO}$ , and ${\text{Φ}}_{NPQ}$ throughout the paper.

In order to reveal the fluorescence characteristics of 225 rice accessions, we evaluated the chlorophyll content (*SPAD*) and eight fluorescence parameters ( $NPQ_{t}$ , ${\text{Φ}}_{II}$ , ${\text{Φ}}_{NO}$ , ${\text{Φ}}_{NPQ}$ , LEF, $F_{v}/F_{m}$ , $q_{L}$ and $q_{P}$ ) in two years (2018 and 2019). **Figure 2A** shows the distribution of each trait. We used the BLUE value to combine the results of the two years, and performed descriptive statistical analysis (**Table S10**). The box plots showed differences (Wilcox test for one group) in fluorescence parameters among the four groups classified by PCA (**Figure 2B**), where $NPQ_{t}$ (P = 0.025), ${\text{Φ}}_{NPQ}$ (P = 0.035), *SPAD* (P = 6.7 × 10^-9^), and $F_{v}/F_{m}$ (P = 0.027) exhibited significant differences among different PCA groups, indicating that the phenotypic differences in *SPAD*, $NPQ_{t}$ , ${\text{Φ}}_{NPQ}$ , and $F_{v}/F_{m}$ among different accessions were affected by the genetic background.

**Figure 3:**
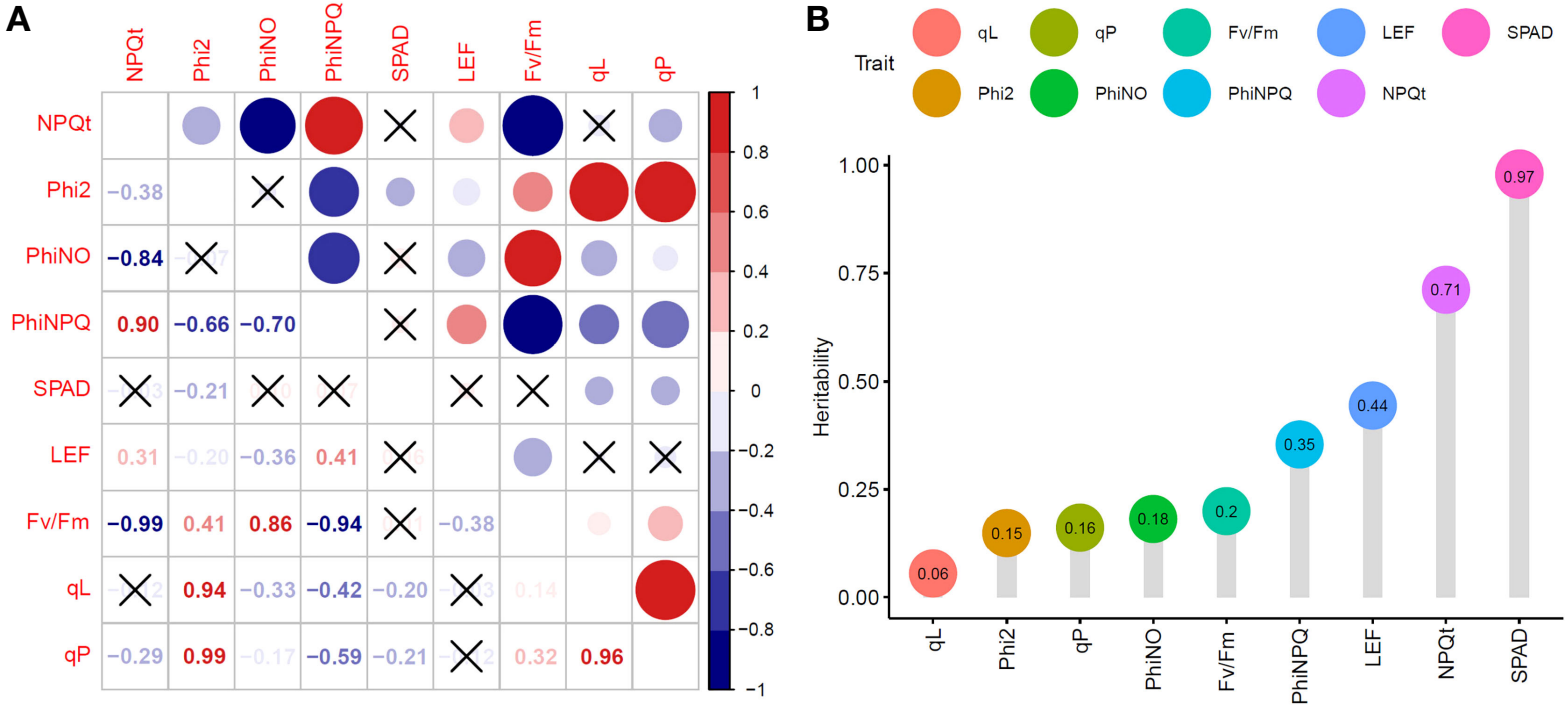
Phenotypic analysis of chlorophyll fluorescence characteristics in 225 rice accessions. **(A)** Correlation analysis of chlorophyll fluorescence characteristics. **(B)** Broad-sense heritability of chlorophyll fluorescence characteristics.

We calculated the Pearson correlation coefficients to investigate the relationship between different fluorescence characteristics. As expected, *SPAD*, an indicator of chlorophyll content, was negatively correlated with ${\text{Φ}}_{II}$ , $q_{L}$ , and $q_{P}$ (R = -0.21, -0.20, -0.21; P< 0.05, respectively). $NPQ_{t}$ , which can reflect non-photochemical quenching, showed a significant positive correlation with ${\text{Φ}}_{NPQ}$ (R = 0.90; P< 0.05). ${\text{Φ}}_{II}$ , an indicator of photochemical efficiency, exhibited significant positive correlations with $F_{v}/F_{m}$ , $q_{L}$ and $q_{P}$ (R = 0.41, 0.94, 0.99; P<0.05, respectively). Additionally, ${\text{Φ}}_{NO}$ showed a significant positive correlation with $F_{v}/F_{m}$ , but negative correlations with ${\text{Φ}}_{NPQ}$ , LEF, $q_{L}$ and $q_{P}$ . LEF exhibited significant positive correlations with $NPQ_{t}$ and ${\text{Φ}}_{NPQ}$ , but negative correlations with ${\text{Φ}}_{II}$ , ${\text{Φ}}_{NO}$ , and $F_{v}/F_{m}$ (**Figure 3A**).

The heritability of traits is a key parameter in breeding selection (Nirmaladevi et al., 2015; Roy and Shil, 2020). Here, *SPAD*, $NPQ_{t}$ , ${\text{Φ}}_{II}$ , ${\text{Φ}}_{NO}$ , ${\text{Φ}}_{NPQ}$ , LEF, $F_{v}/F_{m}$ , $q_{L}$ , and $q_{P}$ exhibited different patterns of heritability, ranging from 0.06 to 0.97 (**Figure 3B**). The heritability of *SPAD*, $NPQ_{t}$ , and LEF was greater than 0.4, while that of ${\text{Φ}}_{NPQ}$ , $F_{v}/F_{m}$ , ${\text{Φ}}_{NO}$ , ${\text{Φ}}_{II}$ , and $q_{L}$ was below 0.4. *SPAD* had the highest heritability of 0.97. These results indicated that *SPAD*, $NPQ_{t}$ , and LEF are greatly influenced by genetic factors.

### 3.3 GWAS and candidate gene search

**Figure 4:**
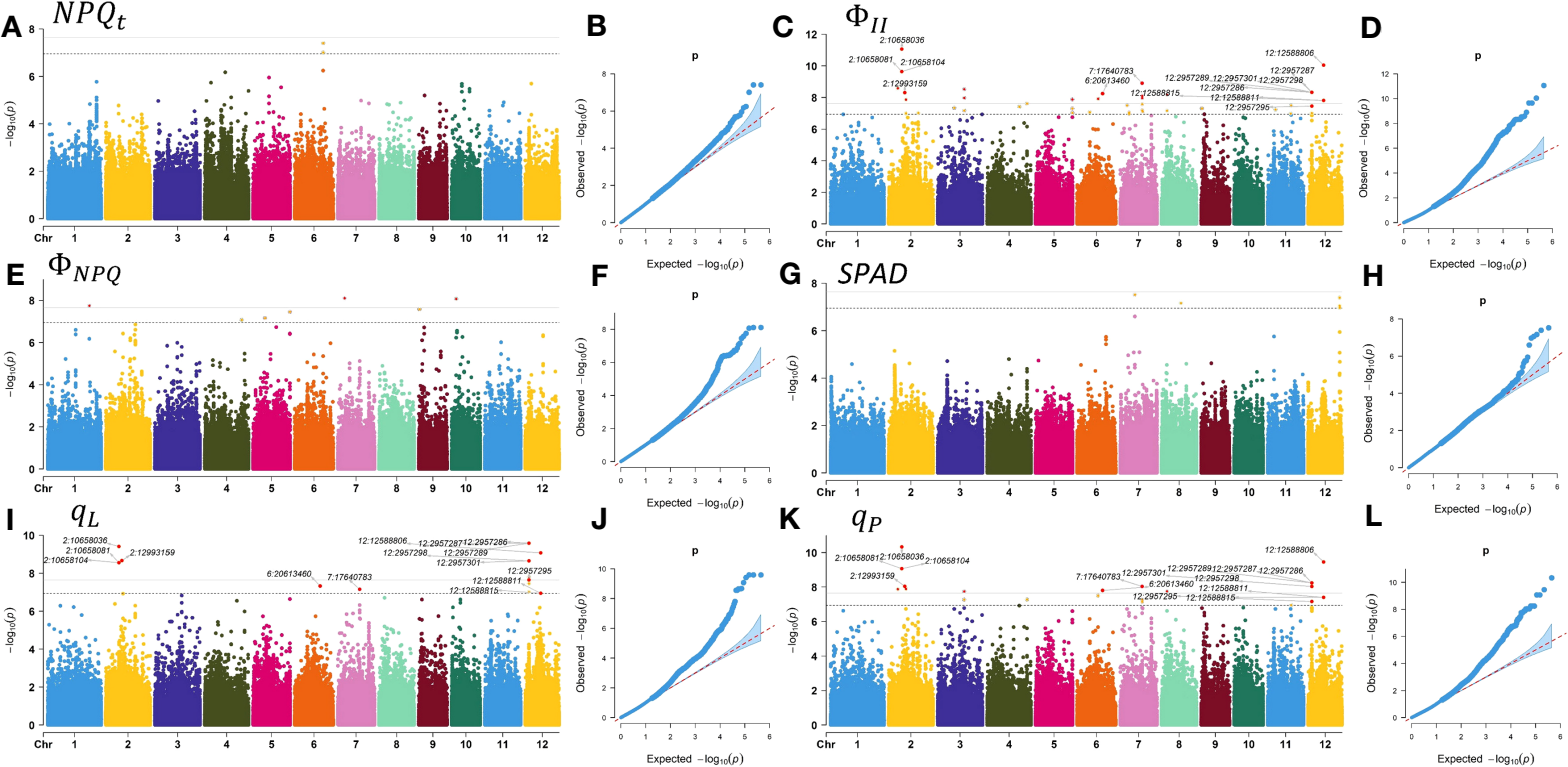
Manhattan plots and quantile-quantile (QQ) plots of GWAS for chlorophyll fluorescence characteristics. **(A)** Manhattan plot for $NPQ_{t}$ . **(B)** QQ plot for $NPQ_{t}$ . **(C)** Manhattan plot for ${\text{Φ}}_{II}$ . **(D)** QQ plot for ${\text{Φ}}_{II}$ . **(E)** Manhattan plot for ${\text{Φ}}_{NPQ}$ . **(F)** QQ plot for ${\text{Φ}}_{NPQ}$ . **(G)** Manhattan plot for the *SPAD*. **(H)** Q-Q plot for the *SPAD*. **(I)** Manhattan plot for $q_{L}$ . **(J)** QQ plot for $q_{L}$ . **(K)** Manhattan plot for $q_{P}$ . **(L)** Q-Q plot for $q_{P}$ .

We conducted a GWAS using the MLM method implemented in GEMMA software and analyzed the final set of 442,634 SNPs. Q and K, which can represent the population structure and kinship, were included in the MLM model to prevent spurious associations, with a significance threshold of P< 1.13 × 10^-7^. By integrating the Manhattan plots for rice chlorophyll fluorescence traits (**Figures 4A–L**) and LD decay rates of 12 chromosomes in 225 rice accessions (**Figure 1C**), and based on the LD coefficient decreasing to half of its maximum at a distance of 1 kb, we selected target intervals at 2 kb upstream and downstream of the SNP, and finally identified 31 significantly associated loci. These loci included 78 SNPs associated with *SPAD*, $NPQ_{t}$ , ${\text{Φ}}_{II}$ , ${\text{Φ}}_{NPQ}$ , $q_{L}$ and $q_{P}$ , which comprised 3, 64, 8, 18, 26, and 5 SNPs, respectively (**Table S5**). Moreover, clear co-localization was observed between ${\text{Φ}}_{II}$ and ${\text{Φ}}_{NPQ}$ and between $q_{L}$ and $q_{P}$ (**Figure S3**), and the co-localization results of ${\text{Φ}}_{II}$ , $q_{L}$ , and $q_{P}$ were annotated in the Manhattan plot (**Figures 4C, I, K**). Based on functional analysis of genes in LD regions, a total of 17 candidate genes were identified for chlorophyll fluorescence characteristics (**Table S6**). No significant SNPs were found for ${\text{Φ}}_{NO}$ , LEF, and $F_{v}/F_{m}$ (**Figure S4**).

### 3.4 Genetic relationship between $\Phi_{II}$ and *SPAD*

**Figure 5:**
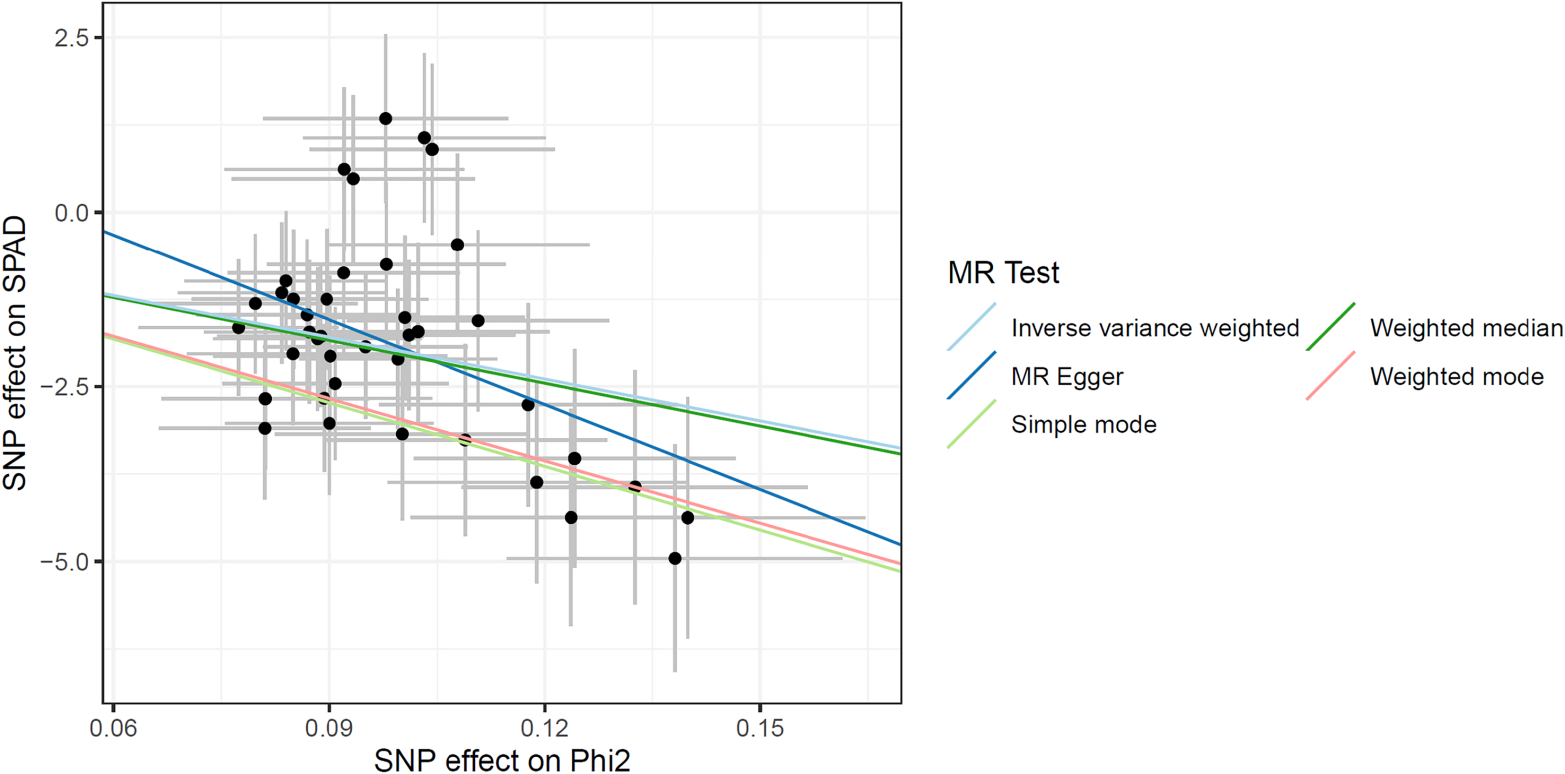
Genetic effects of ${\text{Φ}}_{II}$ and *SPAD* obtained by MR analysis.

In section 3.2, we observed a negative correlation between ${\text{Φ}}_{II}$ and *SPAD*. To comply with the requirements of MR analysis, we included 65 ${\text{Φ}}_{II}$ loci that reached genome-wide significance (P< 1.13 × 10^-7^) in the GWAS analysis. These loci, which exhibited negative genetic effects on *SPAD*, were consistently observed across five analytical methods (**Figure 5**): MR Egger (Beta = -40.47; P< 0.05), Weighted Median (Beta = -20.43; P< 0.05), Inverse Variance Weighted (Beta = -19.94; P< 0.05), Simple Mode (Beta = -30.35; P< 0.05), and Weighted Mode (Beta = -29.69; P< 0.05) (**Table S8**). In sensitivity analysis, homogeneity statistics showed that the effect sizes of the studied loci were homogeneous in MR Egger (P< 0.05) and Inverse Variance Weighted (P< 0.05) methods (**Table S8**). As the Horizontal pleiotropy analysis result was insignificant (intercept = 2.10; P > 0.05) (**Table S8**), we conducted a leave-one-out analysis on the 65 SNPs (**Table S9**). The results further confirmed the negative effect of ${\text{Φ}}_{II}$ on *SPAD*.

### 3.5 Transcriptome analysis of two rice varieties with significant differences in chlorophyll fluorescence characteristics

**Figure 6:**
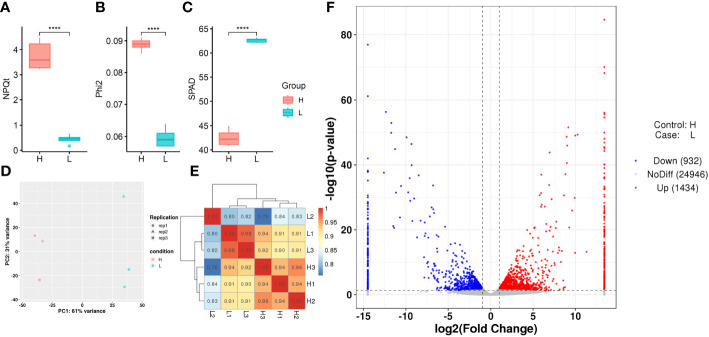
Transcriptome analysis of differentially expressed genes (DEGs) in various samples. **(A, B, C)** $NPQ_{t}$ , ${\text{Φ}}_{II}$ , and *SPAD* of H and L samples using boxplots. **(D)** Principal component analysis (PCA) of H and L samples. **(E)** Correlation test results of H and L samples. **(F)** Volcano plot of DEGs for H and L samples.

To further investigate the genetic basis for chlorophyll fluorescence characteristics in rice, two rice varieties with significant differences in ${\text{Φ}}_{II}$ and *SPAD*, namely D062 (High ${\text{Φ}}_{II}$ type, H) and D133 (Low ${\text{Φ}}_{II}$ type, L), were selected from the population for further analysis. The ${\text{Φ}}_{II}$ and *SPAD* of H and L are presented in **Figures 6A–C**. We collected flag leaves at the heading stage for RNA sequencing with three biological replicates for each accession. Finally, a total of 263,471,192 reads with a Q30 score of 92.33% were generated. Among these reads, 3.70–4.38% were multiply mapped, while 95.62–96.30% were uniquely mapped to the reference genome (**Table S7**). To evaluate the data reliability, correlation analysis (**Figure 6D**) and cluster analysis (**Figure 6E**) were conducted. DEGs between the two varieties (H and L) were identified, including 1,434 upregulated genes and 932 downregulated genes (**Figure 6F**).

**Figure 7:**
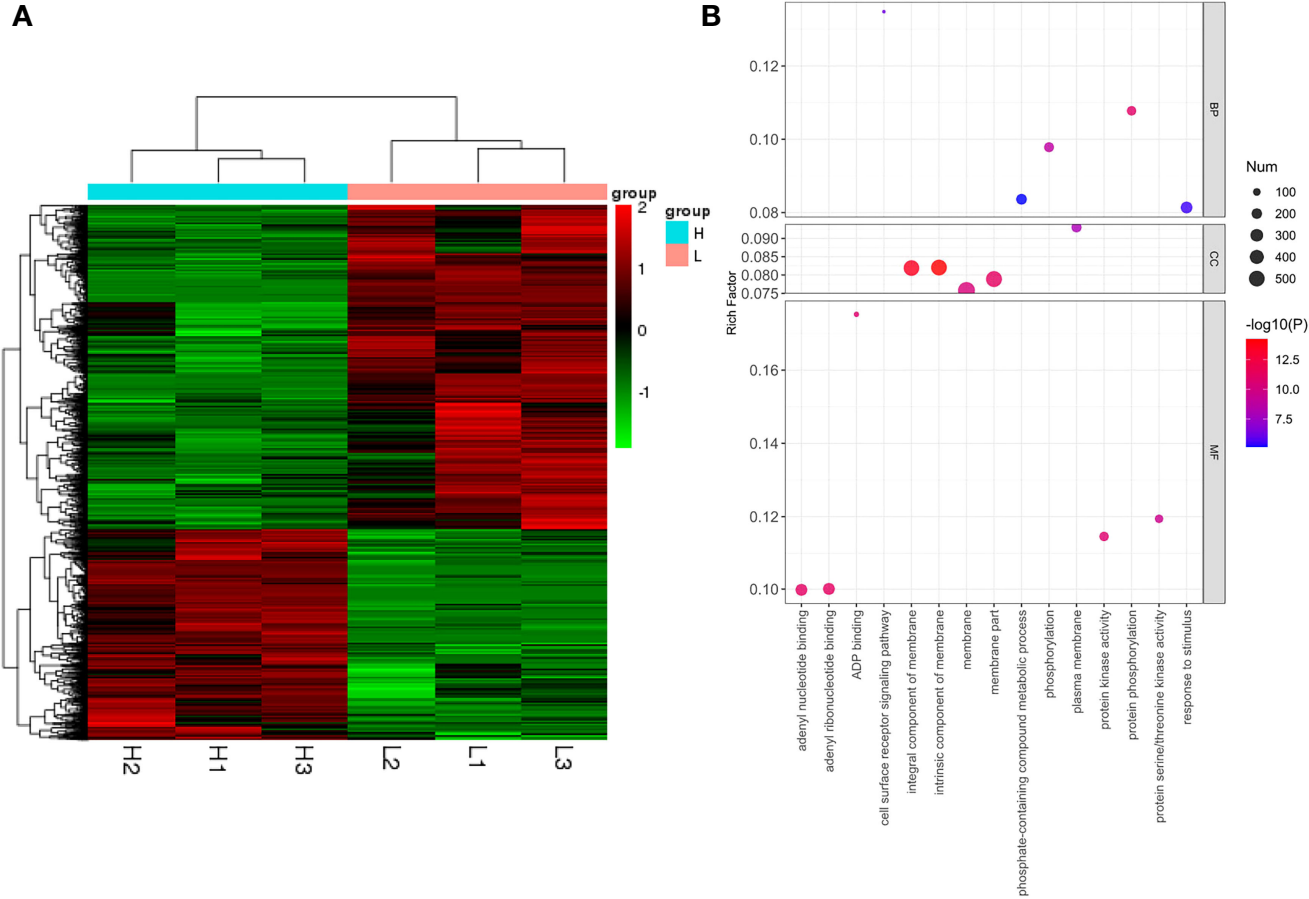
Clustering analysis of differentially expressed genes (DEGs) (H *vs.* L) **(A)**. Bubble chart for GO enrichment analysis of DEGs in H and L **(B)**.

Expression clustering can identify the unknown biological connections between genes. Both H and L type had good correlations within the group, indicating that DEGs in different groups may have specific connections with certain biological processes, metabolisms, and signaling pathways (**Figure 7A**). To uncover the functions of 2,366 DEGs, Gene Ontology (GO) enrichment analysis was conducted, and the DEGs were classified based on their molecular function (MF), biological process (BP), and cellular component (CC). The top five GO terms with the smallest p-values, namely the most significant enrichments, were selected and presented for each category. For the MF category, the top five enriched GO terms were protein phosphorylation (GO:0006468), phosphorylation (GO:0016310), adenyl ribonucleotide binding (GO:0032559), adenyl nucleotide binding (GO:0030554), and protein kinase activity (GO:0004672). Based on the analysis results, these DEGs were likely involved in a host of biochemical reactions necessary for kinase activity, nucleotide binding, and phosphorylation (**Figure 6C**). For the BP category, the top five enriched GO terms were cell surface receptor signaling pathway (GO:0007166), response to stimulus (GO:0050896), protein phosphorylation (GO:0006468), phosphate-containing compound metabolic process (GO:0006796), and phosphorylation (GO:0016310). The results suggested that these DEGs may have crucial functions in cellular signaling, response to environmental stimuli, and metabolic processes (**Figure 6C**). For the CC category, the top five enriched GO terms were intrinsic component of membrane (GO:0031224), integral component of membrane (GO:0016021), plasma membrane (GO:0005886), membrane part (GO:0044425), and membrane (GO:0016020), indicating that these DEGs may be involved in various cellular membrane-related functions (**Figure 7B**). Further scrutiny of the DEGs indicated their potential involvement in regulating the photosynthetic performance of rice, including $NPQ_{t}$ , ${\text{Φ}}_{II}$ , and *SPAD*. Therefore, these genes represent valuable research targets for further investigation and potential avenues for crop improvement.

### 3.6 Discovery of candidate genes for chlorophyll fluorescence characteristics by integrating GWAS and transcriptome data

To further confirm the candidate genes, we validated the genes related to rice chlorophyll fluorescence characteristics by combining GWAS significant regions, LD decay, DEGs, and gene annotation. The *Os03g0583000* and *Os06g0587200* genes were found to be located on the SNPs identified in the GWAS, and showed significant differences in expression levels (|L2FC| > 1, P< 0.05) between the two rice varieties. Therefore, these two genes were considered as the most likely candidate genes. Blast2GO annotations revealed that *Os03g0583000* was a peroxisomal protein, and *Os06g0587200* was a protein kinase containing a catalytic domain (**Table 1**).

**Table 1:** Discovery of candidate genes by integrating GWAS and transcriptome data.

Trait	Gene ID	SNP	L2FC	P-value	Description
${\text{Φ}}_{II}$ , $q_{P}$	*Os03g0583000*	3:21458176	1.40	0.046	Peroxisomal protein
$NPQ_{t}$	*Os06g0587200*	6:23005336	-1.52	0.048	Protein kinase, catalytic domain-containing protein

**Figure 8:**
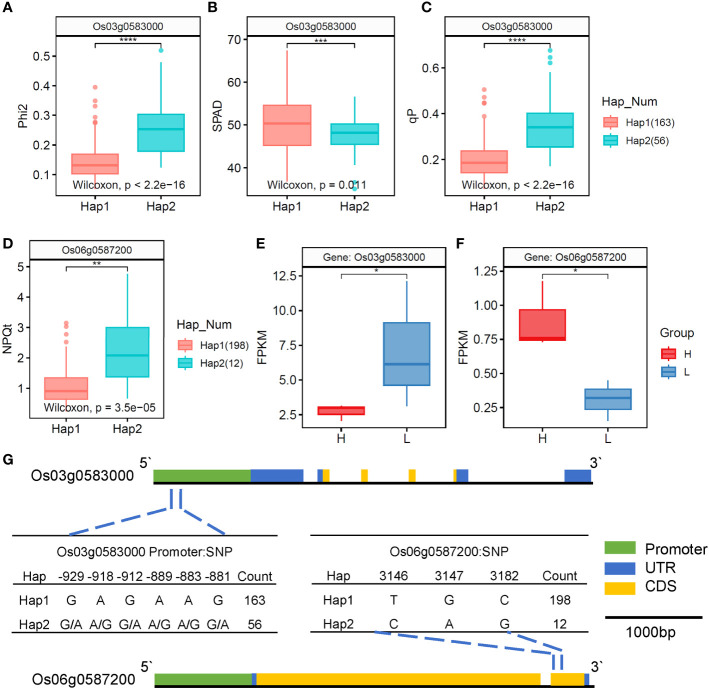
Candidate genes for ${\text{Φ}}_{II}$ , $q_{P}$ , and $NPQ_{t}$ underlying the associated loci on chromosome 03 and chromosome 06. **(A–C)** Boxplots for ${\text{Φ}}_{II}$ , *SPAD*, and $q_{P}$ based on the genotypes. **(D)** Boxplots for $NPQ_{t}$ based on the genotypes. **(E, F)** FPKM values of *Os03g0583000* and *Os06g0587200* in the flag leaves of rice. **(G)** Mutation haplotype analysis and gene region of *Os03g0583000* and *Os06g0587200*. *, **, ***, and **** represent significance levels of 0.05, 0.01, 0.001, and 0.0001, respectively.

We plotted the genetic structure of the two candidate genes (**Figure 8G**), and haplotype analysis showed that six SNPs in the promoter of *Os03g0583000* formed two haplotypes (**Figure 8G**). The inbred lines carrying haplotype 1 had significantly lower ${\text{Φ}}_{II}$ and $q_{P}$ values while significantly higher *SPAD* values than those carrying haplotype 2 (**Figures 8A–C**). In addition, transcriptome analysis showed that the H type had a significantly lower FPKM value of *Os03g0583000* than the L type (**Figure 8E**), indicating that *Os03g0583000* was the most likely candidate gene for ${\text{Φ}}_{II}$ and $q_{P}$ . The genetic variations at the identified SNP loci were also found to affect *SPAD*. In addition, three SNPs in the exon region of *Os06g0587200* formed two haplotypes (**Figure 8G**). The inbred lines carrying haplotype 1 had significantly lower $NPQ_{t}$ than those carrying haplotype 2 (**Figure 8D**). Transcriptome analysis showed that the H type had a significantly higher FPKM value of *Os06g0587200* than the L type (**Figure 8F**), and the H type belonged to haplotype 2, whereas the L type belonged to haplotype 1, indicating that *Os06g0587200* was the most likely candidate gene for $NPQ_{t}$ .

## 4 Discussion

Chlorophyll is the primary light-harvesting pigment as well as the reaction center that directly influences light interception and conversion in plants, thereby affecting plant photosynthetic capacity and crop productivity (Croft et al., 2017). Chlorophyll fluorescence is an important indicator to reflect the photosynthetic status of plants. Therefore, chlorophyll fluorescence parameters are highly effective and widely used indicators for studying photosynthesis (Ripoll et al., 2016). Previous studies have demonstrated that the chlorophyll content of mature rice flag leaves is significantly correlated with the Rubisco content, total photosynthesis rate, and maximum quantum yield of photosystem II ( $F_{v}/F_{m}$ ) (Kumagai et al., 2009). It has been reported that plants in optimal photosynthetic states usually exhibit higher yields and better growth due to their superior ability to utilize solar energy, which can increase their energy levels and nutrient efficiency (Yin and Struik, 2015; Gu et al., 2017). Therefore, this study investigated the chlorophyll fluorescence characteristics of rice, including chlorophyll content (*SPAD*) and eight chlorophyll fluorescence parameters ( $NPQ_{t}$ , ${\text{Φ}}_{II}$ , ${\text{Φ}}_{NO}$ , ${\text{Φ}}_{NPQ}$ , LEF, $F_{v}/F_{m}$ , $q_{L}$ and $q_{P}$ ), and combined differential expression analysis, correlation and heritability analysis to determine the relationships between various fluorescence characteristics at the phenotype level. *SPAD*, an indicator of chlorophyll content, was negatively associated with ${\text{Φ}}_{II}$ (R = -0.21, P< 0.05), which was further validated by MR analysis at the genetic level. The relationship between *SPAD* and ${\text{Φ}}_{II}$ has been examined in many studies. Trachsel et al. (2010) observed a positive correlation, and conversely Song et al. (2018) found a non-significant negative correlation between them. In contrast, Fu et al. (2013) identified no significant correlation. Notably, Singh et al. (2019) employed a second-order polynomial function to model the regression relationship between *SPAD* and ${\text{Φ}}_{II}$ . These results suggested the presence of a complex nonlinear relationship between *SPAD* and ${\text{Φ}}_{II}$ across different materials and growth stages. In this study, an analysis of 225 rice accessions at the heading stage (a relatively mature developmental stage) revealed a weak negative correlation between *SPAD* and ${\text{Φ}}_{II}$ in flag leaves. *SPAD* is generally measured based on the unit leaf area, and can only reflect the chlorophyll content per unit leaf area (Uddling et al., 2007). Previous research has indicated a positive correlation between *SPAD* and leaf thickness (Li et al., 2009). In leaves with higher thickness, despite a higher chlorophyll content, reduced translucency limits the optimal utilization of each chlorophyll molecule, leading to a negative correlation between *SPAD* and ${\text{Φ}}_{II}$ . In this scenario, the leaves with lower *SPAD* and higher ${\text{Φ}}_{II}$ levels may have higher light use efficiency in plants. Further investigation is required to better understand the correlation between *SPAD* and ${\text{Φ}}_{II}$ .

In this study, we used 632.17 Gb of high-quality sequencing data to identify 9,989,556 SNP loci through a comparison with the reference genome. The high density of markers allowed a more detailed GWAS analysis of chlorophyll fluorescence characteristics, facilitating a more complete identification of candidate genes related to chlorophyll content and fluorescence parameters. As a result, 17 candidate genes were identified to be associated with $NPQ_{t}$ , ${\text{Φ}}_{NPQ}$ , *SPAD*, $q_{L}$ and $q_{P}$ , which are distributed on chromosomes 1, 2, 3, 4, 6, 7, 8, 10, and 12. Compared with traditional QTL mapping methods, GWAS provided a higher resolution for identifying candidate genes. To investigate the genetic basis for different fluorescence characteristics in rice, we performed transcriptome sequencing of two rice varieties (H and L) with significant differences in ${\text{Φ}}_{II}$ and *SPAD*. We identified 2,366 DEGs and analyzed their functions through GO enrichment analysis. The phosphorylation-related pathways, including protein phosphorylation (GO:0006468), phosphorylation (GO:0016310), protein kinase activity (GO:0004672), and phosphate-containing compound metabolic process (GO:0006796), play important roles in photosynthesis because photosynthesis involves a large number of protein phosphorylation reactions (Allen, 1992). In addition, the membrane-related pathways, including intrinsic component of membrane (GO:0031224), integral component of membrane (GO:0016021), plasma membrane (GO:0005886), membrane part (GO:0044425), and membrane (GO:0016020), are also important in photosynthesis because it occurs in chloroplasts, which have many important membrane structures. A large number of membrane proteins are embedded in these structures. Previous studies have demonstrated that membrane fluidity is significantly correlated with Rubisco activase and net photosynthesis (Kim and Portis, 2005). Exogenous substances such as polyamines can decrease the membrane oxidation damage, contributing to improvement of photosynthesis (Farooq et al., 2009).

Finally, by combining GWAS analysis, transcriptome analysis, gene annotation, GO analysis, and haplotype analysis of flag leaves, we identified the most likely candidate genes. Blast2GO predicted that *Os03g0583000* is a peroxisomal protein and has significant correlations with ${\text{Φ}}_{II}$ , $q_{P}$ and *SPAD*. Its FPKM value in the H type was significantly lower than that in the L type. *Os06g0587200* was annotated by annovar and predicted by Blast2GO to contain a protein kinase catalytic domain. Its haplotypes showed significant correlations with $NPQ_{t}$ , and its FPKM value in the H type was also significantly higher than that in the L type. Furthermore, *Os03g0583000* and *Os06g0587200* are both involved in the membrane (GO:0016020) and integral component of membrane (GO:0016021) pathways. Peroxisomal APX and CAT have been shown to be associated with enzyme activity during photoprotection in rice plants (Sousa et al., 2019), and peroxisomes and mitochondria can coordinately regulate NAD^+^ transport protein activity to enhance photosynthesis and seed yield under high CO_2_ levels (Feitosa-Araujo et al., 2022). In Arabidopsis, imaging analysis of fluorescence also showed that peroxisomes are involved in the response of fluorescence parameters to drought stress (Li and Hu, 2015). In addition, Zhang et al. (2016) found that *OsAld-Y* is localized in the peroxisome and participates in photosynthesis by affecting leaf photosynthesis rate and sugar metabolism, which contribute to chlorophyll accumulation, chloroplast development, and plant growth. Therefore, in this study, *Os03g0583000* and *Os06g0587200* may participate in peroxisome-related antioxidant and photoprotection processes as well as chlorophyll synthesis processes to regulate plant chlorophyll fluorescence characteristics.

## 5 Conclusion

This study conducted a genome-wide association study (GWAS) on 225 rice accessions. In the phenotypic and Mendelian randomization (MR) analysis, a weak negative correlation was observed between the chlorophyll content and actual quantum yield of photosystem II ( ${\text{Φ}}_{II}$ ). The phenotypic diversity observed in *SPAD*, $NPQ_{t}$ , ${\text{Φ}}_{NPQ}$ , and $F_{v}/F_{m}$ among accessions was affected by genetic background. Furthermore, the GWAS identified 78 SNPs and 17 candidate genes significantly associated with *SPAD*, $NPQ_{t}$ , ${\text{Φ}}_{II}$ , ${\text{Φ}}_{NPQ}$ , $q_{L}$ and $q_{P}$ . Additionally, by transcriptome analysis, we identified the key genes and pathways responsible for the differences in ${\text{Φ}}_{II}$ , $q_{P},\;$ and *SPAD* between two representative rice varieties, and combined GWAS with transcriptome analysis suggested that two candidate genes (*Os03g0583000* from ${\text{Φ}}_{II}$ & $q_{P}$ traits and *Os06g0587200* from $NPQ_{t}$ trait), which are respectively associated with peroxisomes and protein kinase catalytic domains, are involved in regulating the chlorophyll content and fluorescence. This study provides novel insights into the correlation among chlorophyll content and fluorescence parameters and the genetic mechanisms in rice, and offers valuable information for the breeding of rice with enhanced photosynthetic efficiency.

## Data availability statement

The datasets presented in this study can be found in online repositories. The names of the repository/repositories and accession number(s) can be found below: https://www.ncbi.nlm.nih.gov/, PRJNA979327.

## Author contributions

JH and DX designed and supervised the research. ZX and JH revised the manuscript. DX, FW and ZZ and guided the content of the article. SL, ZX and YW investigated phenotypic of chlorophyll fluorescence characteristics. SL and YW collected the sample for RNA-seq. SL and YW performed the data analysis. SL finished the writing of the manuscript. All authors contributed to the article and approved the submitted version.
