# Supplementary material for: Exploration of chlorophyll fluorescence characteristics gene regulatory in rice (Oryza sativa L.): a genome-wide association study
Source: Front Plant Sci. 2023 Sep 7;14:1234866. doi: 10.3389/fpls.2023.1234866 (PMC10513790; doi:10.3389/fpls.2023.1234866)
Supplement: Supplementary file 1 [file DataSheet_1.pdf]

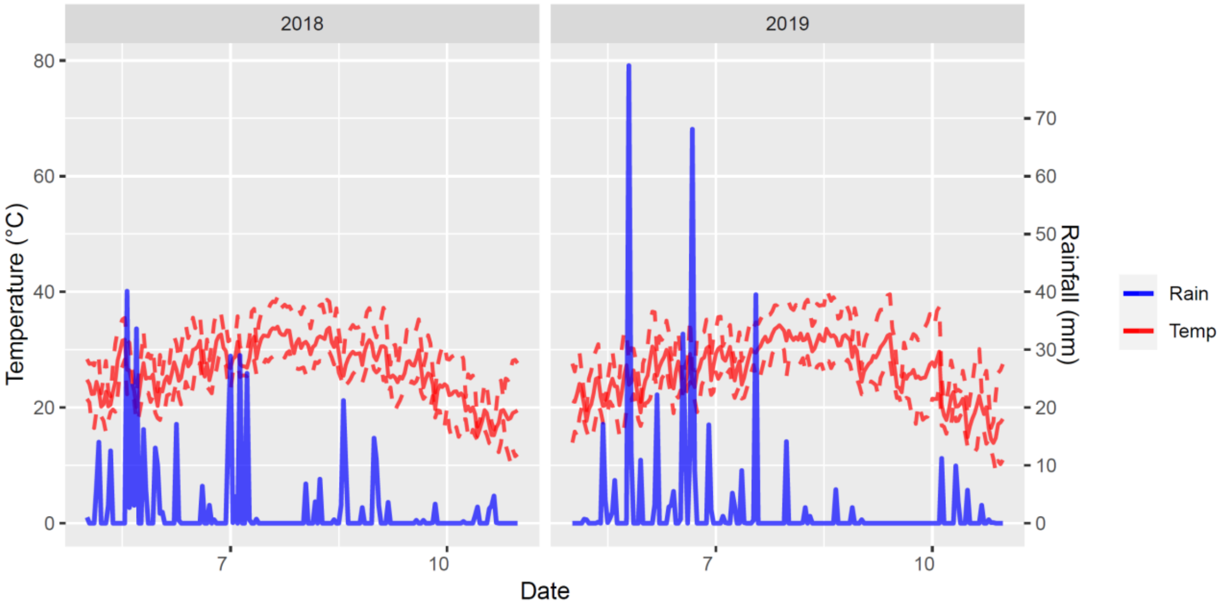

Figure S1 Changes in daily temperature and precipitation during the whole growth period of rice under natural conditions

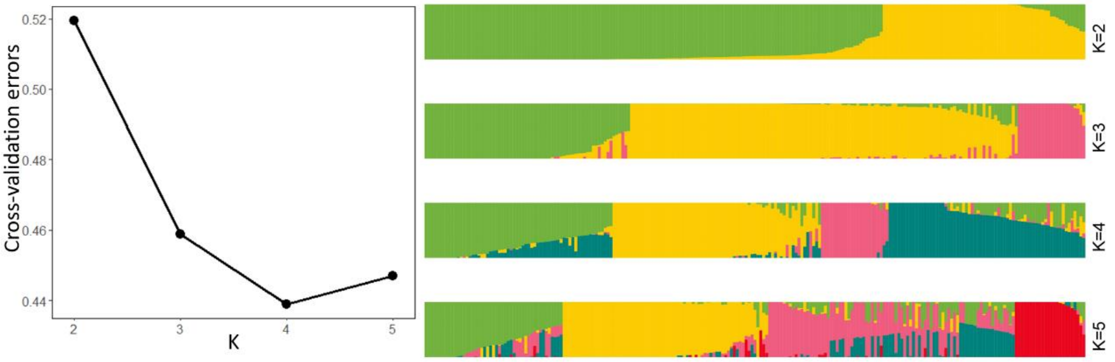

Figure S2 (A) Cross-validation errors plot in the population. (B) Membership fractions of the genotypes in sub-populations (K = 2, 3, 4, 5) inferred

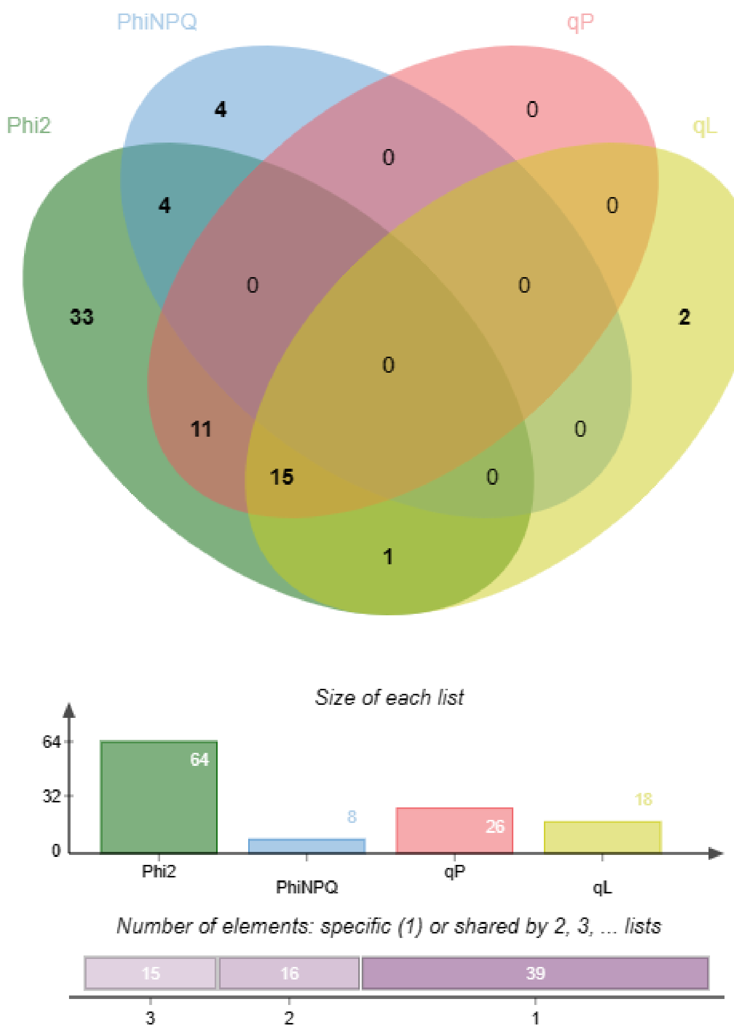

Figure S3 GWAS colocalization of Phi2, PhiNPQ, qP and qL

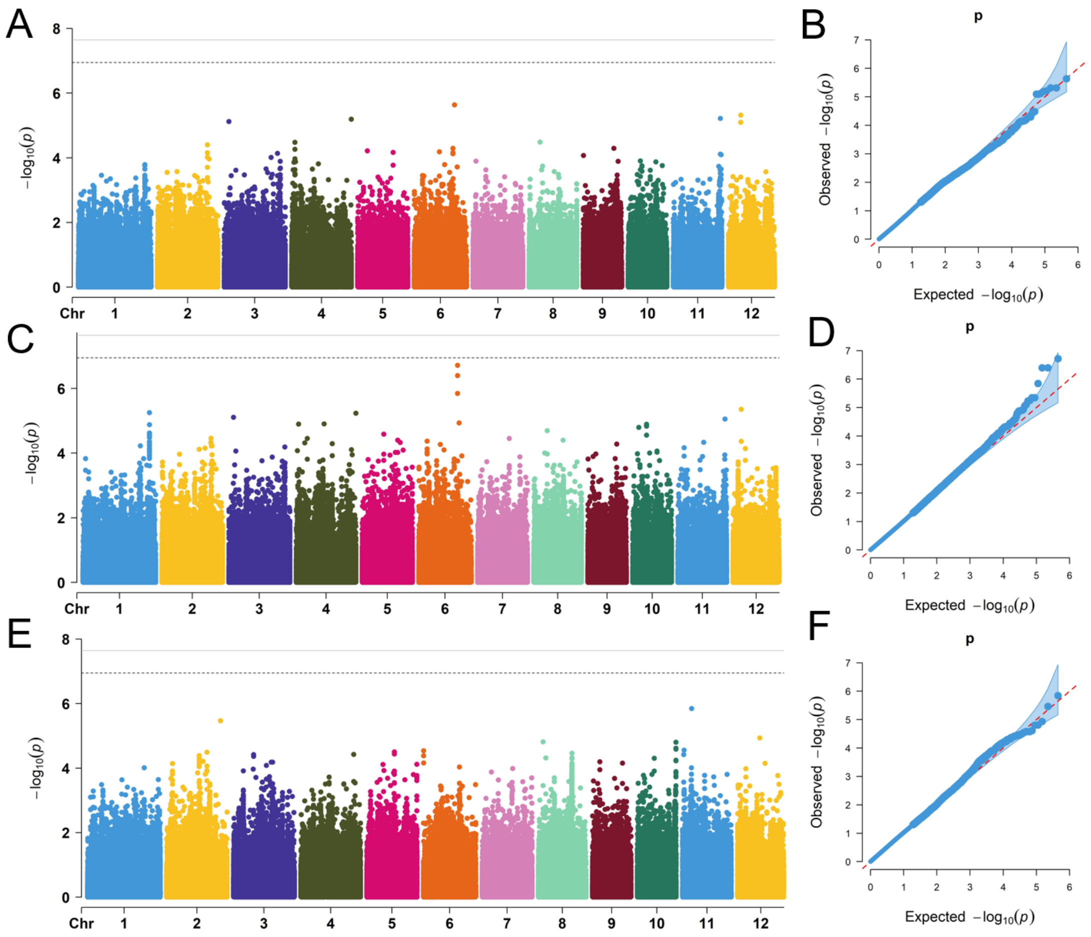

Figure S4 Manhattan plots and quantile-quantile (Q-Q) plots of genome-wide association studies for PhiNO (A,B), LEF (C,D) and Fv/Fm (E,F)
